# The importance of genetic counselling and testing in inherited eye diseases: A population-based retrospective study

**DOI:** 10.1371/journal.pone.0318492

**Published:** 2025-02-13

**Authors:** Michal Kaminer Abargel, Michal Macarov, Karen Hendler, Claudia Yahalom

**Affiliations:** 1 Faculty of Medicine, Hebrew University of Jerusalem, Jerusalem, Israel; 2 Department of Ophthalmology, Hadassah Medical Center, Jerusalem, Israel; University College London Institute of Ophthalmology, UNITED KINGDOM OF GREAT BRITAIN AND NORTHERN IRELAND

## Abstract

**Purpose:**

Inherited eye diseases (IEDs) are among the main causes of visual impairment and blindness in children and young people worldwide. The objective of our study was to characterize the prevalence and distribution of the most common IEDs and causative genes in our population.

**Study design:**

Retrospective study based on medical records of patients with IEDs who underwent genetic counselling through our multidisciplinary low vision center from 2018 to 2020.

**Methods:**

Data retrieved from medical files included: year of consultation, age, gender, ethnicity, diagnosis, gene variants and mode of inheritance.

**Results:**

228 patients were included in our study. The most common diagnoses were inherited retinal diseases (IRDs) (41.2%) and albinism (32%). In 2018 and 2019 the number of patients reaching out for genetic counselling was between 50 and 60; this number doubled by 2020. The rate of pathogenic variant detection was 65.3%. The most common genes identified were *TYR* (29.2%)*, OCA2* (7.9%)*, ABCA4 (5.3%), TRPM1* (5.3%) and *USH2A* (4.4%).

**Conclusion:**

Genetic counselling and testing became an essential part of caregiving for patients and families affected by these severe IEDs. The most common IEDs were IRDs in the Muslim population and albinism in the Jewish population. Pathogenic variants in the *TYR* gene were the most common in our cohort, *OCA2* gene was the second in frequency, followed by *ABCA4*, *TRPM1* and *USH2A* genes. We detected an increasing trend over the studied time in the number of patients reaching out for genetic counselling.

## Introduction

Inherited eye diseases (IEDs) include numerous conditions such as developmental eye abnormalities, retinal and corneal degenerations, congenital cataract, and hereditary neurodegenerative disorders of the optic nerve [1]. They may appear as isolated eye pathology or as part of a syndrome along with additional systemic features.

### The prevalence of IEDs

is around 1 in 1,000 individuals worldwide [1,2]. They are one of the most common causes of visual impairment and blindness in young people around the world [3,4]. In African countries, IEDs are responsible for around 14% of childhood blindness, while in developed countries, such as Europe and the United States, they account for approximately 53% [3,5–9].

Inherited retinal diseases (IRDs) and albinism are among the most common IEDs causing visual impairment [4].

IRDs are a large group of both progressive and non-progressive conditions characterized by retinal degeneration [1]. Peripheral vision loss and night blindness are typical presentations for rod dominant dystrophies, such as retinitis pigmentosa. Whereas, central vision loss, impaired color perception, photophobia, and nystagmus are classic manifestations of cone dominant dystrophies, like Stargardt disease, macular degeneration and cone-rod dystrophy [10–12].

Oculocutaneous albinism (OCA) is an autosomal recessive disorder characterized by an absolute or partial deficiency in the production of melanin in the melanocytic cells, causing decreased pigmentation of the hair, skin, and eyes, ranging from mild lack of pigment to complete depigmentation [13–16]. The lack of melanin during the embryonic development is associated with ocular anomalies, including foveal hypoplasia, retinal and iris hypopigmentation, thinning of optic nerve fibers, and high refractive errors. Affected patients show a wide range of visual impairment, nystagmus, and photophobia [17].

Albinism can also manifest as a part of a syndrome or exclusively in ocular forms [13,14,18,19]. Most common OCA types include OCA1 responsible for 50% of all cases worldwide, and most common in Caucasians [13,20] and OCA2 accounting for 30% of cases worldwide and most found in African population [13,21].

Indications for genetic counselling and testing include reaching a specific diagnosis, disease prognosis, family planning, treatment options and research purposes. Another important aspect of genetic testing is the psychological comfort associated with the fact of “doing something” instead of staying focused in a hopeless diagnosis with almost no treatment available to restore vision [2]. Genetic counselling results in a family understanding that there is something they can do for future family planning regarding a specific disease.

The aim of our study was to analyze the genetic counselling sessions done though our low vision center, to investigate the most common IEDs and causal genes seen in our population, and to examine whether there has been a trend in the number of inquiries for genetic counselling over the studied years. In addition, we aimed to evaluate the rate of causal gene detection.

## Materials and methods

This was a retrospective study of patients who underwent genetic counselling through our specialized low vision clinic located in a tertiary hospital. The clinic (a national referral center for patients with low vision) has a multidisciplinary team that includes pediatric ophthalmologists, low vision optometrists, a social worker and genetic counsellors. All patients with suspected IEDs are routinely advised to perform genetic counselling and testing. Generally, most of the referred patients choose to undergo genetic counselling sessions, where they get detailed information about the suspected disease inheritance and options for family planning. Following pedigree collection and genetic counselling, patients take a personal decision whether to perform genetic testing, with a majority opting to complete advised molecular testing.

Medical records of 228 probands referred with a clinical diagnosis of IEDs, who underwent genetic counselling from January 2018 until December 2020 were retrieved. Data were accessed for research purposes from January 16^th^ 2023 to March 4^th^ 2023.

The study adhered to the tenets of Helsinki and was approved by the Institutional Review Board at Hadassah-Hebrew University Medical Center, Jerusalem. The need for consent was waived by the ethics committee. Data were fully anonymized for analysis.

Clinical and genetic data were drawn from the patient’s medical records, including diagnosis, ethnicity, type of genetic testing performed, gene variants identified and mode of inheritance.

Statistical analyses were performed by using the statistical package for social sciences software version 27 (SPSS Inc., Chicago, IL, USA). P Value of 0.05 or less was considered statistically significant. To examine the relationship between two categorical variables Chi-square and Fisher’s exact test were used. To compare a quantitative variable between two groups the t-test was used.

## Results

During the studied period 228 probands who underwent genetic counselling because of a clinical diagnosis of an IED were included in this study, of whom 133 (58.3%) were male and 95 (41.6%) were female.

Regarding ethnicity, 164 (72.3%) patients were Jewish, 55 (24.1%) patients were Muslims, 9 (3.9%) patients categorized as “others” (Table 1).

**Table 1:** Demographics of studied population.

		Years
**Age at consultation**	Mean	18.19
	Median	12
		Number (%)
**Gender**	Male	133 (58.3)
	Female	95 (41.6)
Total		228 (100.0)
**Ethnicity**	Jewish	164 (72.3)
	Muslim	55 (24.1)
	Other	9 (3.9)
**Parents consanguinity**	No	171 (75)
	Yes, 1^st^ Cousins	43 (18.9)
	Yes, 2nd Cousins or more distant	14 (6.1)
**Genetically tested**	Yes	173 (75.9)
	Molecularly solved cases	113 (65.3%)
**Year of genetic counselling**	2018	51 (22.4)
	2019	59 (25.9)
	2020	118 (51.8)
**Genetic test used**	Sanger	93 (50.0)
	WES	42 (22.58)
	Panel	34 (18.28)
	Other	17 (9.14)

The mean age of the cohort was 18.19 (SD 19.32, range 0–78 years).

In 171 (75%) patients, parents were non-consanguineous, in 43 (18.8%) patients parents were first cousins, and in 14 (6.1%) patients, parents were second cousin or more distant (Table 1). Following genetic counselling, 173 (75.9%) patients proceeded to genetic testing. Total number of genetic counselling sessions increased during the studied years (Table 1).

Molecular testing was performed using several techniques, mainly Sanger and whole exome sequencing (Table 1).

**Fig 1:**
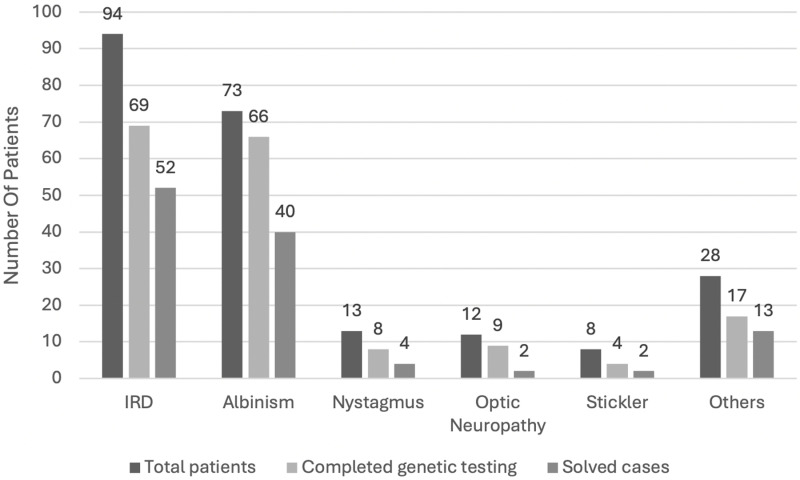
Inherited eye diseases distribution and solved rate among studied patients.

Definitive molecular diagnosis was reached in 113 patients, with a pathogenic variants detection rate of 65.3% during all studied years. Solved cases were based in pathogenic or likely pathogenic variants only. Modes of inheritance in solved cases were autosomal recessive in 88, autosomal dominant in 12 and X-linked in 13 cases. Phasing was done when parents were available for testing. Main diagnoses for IEDs included inherited retinal dystrophies in 94 patients, albinism in 73 patients, infantile nystagmus in 13 patients, optic neuropathy in 12 patients and others (Fig 1).

“Other diseases” grouped all other diagnoses with less than 10 patients per diagnosis and included: congenital cataract, aniridia, Axenfeld-Rieger syndrome, Fhonda, retinoschisis and others.

Among patients with IRDs, genetic testing was completed in 69 patients and the causative gene was found in 52 of them (75.3% detection rate). In 66 patients with albinism that performed genetic testing, the causative gene was identified in 40 cases (60.6% detection rate). In infantile nystagmus, the gene was identified in 4/8 patients who underwent genetic testing (50% detection rate) (Fig 1).

**Fig 2:**
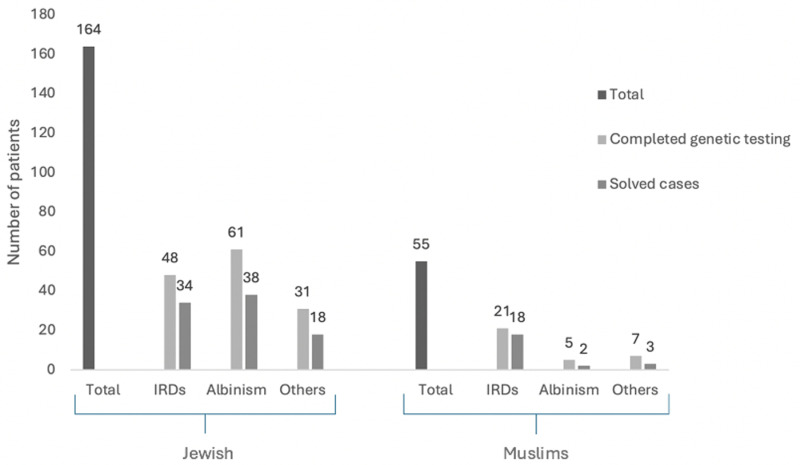
Main diagnosis and solved rate according to ethnicity.

Main disease distribution varied according to ethnicity. Among the Jewish population, the main cause was albinism (38.0%) while in the Muslim population, 60% individuals were diagnosed with retinal dystrophies. The difference was statistically significant (value < 0.001 (chi-square test) (Fig 2).

Main identified genes were *TYR*, *OCA2*, *ABCA4* and *TRPM1*, with a varying distribution in relation to ethnicity (Table 2).

**Table 2:** Distribution of main genes identified related to ethnicity.

Gene	Total number of patients (%)	Jewish	Muslims	Others
*TYR*	33 (29.2%)	31	2	
*OCA2*	9 (7.9%)	8	1	
*ABCA4*	6 (5.3%)	2	3	1
*TRPM1*	6 (5.3%)	4	2	
*UHS2A*	5 (4.4%)	4	1	
*CNGA3*	4 (3.5%)	0	4	
*FRMD7*	4 (3.5%)	4	0	
*RPGR*	3 (2.6)	2	1	

Only genetically solved cases were considered.

Others: genes that were identified in less than 3 patients.

## Discussion

During the last decade, genetic counselling and testing became an integral part of patient’s caregiving in ophthalmology, moving from a “research tool” towards a significant clinical service for affected families. The information given during the counselling session, helps patients to understand the transmission of IEDs and possibilities of prevention [2]

A clear molecular diagnosis not only provides information to the affected family on the disease’s course and expected prognosis, but also aids in their “family planning” decisions [1]

Currently prenatal genetic diagnosis and preimplantation genetic testing (PGT) serve as key strategies to prevent recurrence of inherited disorders in families affected with IEDs. The successful use of PGT have been reported for several IEDs, as a permitted option for preventing diseases known to cause blindness or severe visual impairment [5].Over the past decades, various methods have been developed to directly identify pathogenic variants, including specific variant screenings, single-gene sequencing, and next-generation sequencing (whole exome sequencing (WES) and multi-gene panels). In our cohort, the main technique used was Sanger sequencing; this high prevalence can be explained due to its frequent use in our setting, to identify pathogenic variants extremely common in different ethnic groups in our population. WES and gene panels for eye diseases were also commonly used in our studied patients.

Despite significant advancements in IEDs molecular diagnosis, treatment options remain limited. In selected cases, genetic therapy may be a viable option, such as Neparvovec for *RPE65-*associated retinal degeneration, as well as other new clinical trials investigating the use of innovative approaches [22,23].

In our cohort, the most common IEDs that led individuals to seek genetic counselling were IRDs and albinism. This is in accordance with a previous study published by our group in 2022 where we concluded that the main causes for visual impairment in children in our population, were albinism and IRDs [4].

Pathogenic variants in the *TYR* gene were the most common in our cohort (29.2%). The significantly high rate of TYR gene as the cause of albinism in our population is due to frequent founder pathogenic variants such as c.1037-7T > A (IVS2-7T > A) and c.140G > A (G47D) in in the Ashkenazi and Moroccan Jews respectively [24].

*ABCA4*, *TRPM1* and *USH2A* genes were the most common identified IRDs genes in our study in accordance with previous studies in our population, reporting that *ABCA4* and *USH2A* genes are the most common genes causing IRDs in Israel [25].

*TRPM1* gene was relatively more frequent in our study when compared to Sharon’s study. We believe that this finding might be related to the relatively young age for genetic testing in our cohort (mean age 18 years), with a relatively higher number of patients with an early onset congenital stationary night blindness phenotype.

Even though all patients with a clinical diagnosis of IEDs are offered to undergo genetic counselling and testing, a small percentage choose not to proceed to complete these tests for personal or religious reasons. There was an increasing trend in the number of patients seeking genetic counselling over the studied years. The increased uptake in genetic counselling for IEDs might be related to improved physicians alertness regarding IEDs prevention and possibility of genetic therapy in specific cases, as well as an increased awareness among patients regarding the availability of these tests. In addition, advanced molecular techniques were progressively introduced to the medical system in our country during the last years and today are completely covered by the public health system, facilitating genetic testing.

Overall, we succeeded to identify the genetic basis of disease in 65.3% of the studied patients, remaining stable during the studied years. Pathogenic variants detection rate was higher in IRDs (75%) compared to albinism (60%) in our study. Chan published in 2023 a gene detection yield for albinism of 66%, with no significant differences between ethnicities, comparable to our results [26].

Regarding IRDs, the reported pathogenic variants identification worldwide, using whole exome sequencing or targeted NGS (i.e., gene panels) and whole genome sequencing, ranges between 50 and 76% [27,28]. The percentage found in our study is within the range of diagnostic rates previously reported in other cohorts of IRDs patients.

Limitations in our study include its retrospective nature, limiting the data available in studied patients. Further prospective follow-up might reveal important data like the use of prenatal diagnosis or PGD in this population.

In conclusion, the importance of genetic counselling in IEDs increased during the last years. It helps affected families to understand the prognosis of the disease and aids in family planning. Learning about the ethnic correlation of certain diseases with main causal genes in different populations, is an important step towards future public heath tactics for screening.

## Supporting information

S1 File Manuscript data. (XLSX)
